# A survey on the challenges, limitations, and opportunities of online testing of infants and young children during the COVID-19 pandemic: using our experiences to improve future practices

**DOI:** 10.3389/fpsyg.2023.1160203

**Published:** 2023-06-13

**Authors:** Montana J. Shore, Danielle L. Bukovsky, Sylvia G. V. Pinheiro, Brendan M. Hancock, Emma M. Liptrot, Valerie A. Kuhlmeier

**Affiliations:** Department of Psychology, Queen’s University, Kingston, ON, Canada

**Keywords:** developmental science, methodology, online testing, virtual studies, COVID-19

## Abstract

In developmental psychology, the widespread adoption of new methods for testing children does not typically occur over a matter of months. Yet, the COVID-19 pandemic and its associated social distancing requirements created a sudden need among many research groups to use a new method with which they had little or no experience: online testing. Here, we report results from a survey of 159 researchers detailing their early experiences with online testing. The survey approach allowed us to create a general picture of the challenges, limitations, and opportunities of online research, and it identified aspects of the methods that have the potential to impact interpretations of findings. We use the survey results to present considerations to improve online research practices.

## Introduction

The declaration of the COVID-19 pandemic in March 2020 introduced restrictions and challenges that altered the research practices of many researchers within the field of psychology. Many academic institutions implemented various strategies such as work-from-home measures, fully online courses, and social distancing to combat the spread of the airborne virus. These strategies made in-person psychological research particularly difficult. As a result, many researchers were prompted to explore alternate methods of testing—most notably, internet-based interactions with participants. Arguably, developmental psychology researchers focused on early childhood felt the pressure of this shift particularly acutely, due to constraints such as laboratory room capacity limits, mask mandates, and the understandable hesitancy of families to engage in in-person research. As evidenced by attendance at online workshops (e.g., BeOnline, 2021, 2022; ICIS, 2020), many developmental scientists moved quickly to learn online testing techniques from the relatively few in our field who had been developing and utilizing new platforms and procedures.

From 2020 to the writing of this paper, researchers have published papers focused on specific aspects of the online testing of infants and children. Most notably, a special issue of *Frontiers in Psychology* in 2021 (*Empirical Research at a Distance: New Methods for Developmental Science*, edited by Amso, Cusack, Oakes, Tsuji, and Kirkham; see Tsuji et al., 2022) has 39 papers documenting individual labs’ use of online testing for research topics that include working memory (e.g., Ross-Sheehy et al., 2021), language (e.g., Ozernov-Palchik et al., 2022), emotion perception (e.g., Yamamoto et al., 2021), object physics (Filion and Sirois, 2021), eating behaviors (e.g., Venkatesh and DeJesus, 2021), number (Silver et al., 2021), theory of mind (Schidelko et al., 2021), and parent–child interaction (Shin et al., 2021). Other papers have focused specifically on a particular testing platform or stimulus-presentation software, including *Zoom* (e.g., Bambha and Casasola, 2021) and *LookIt* (e.g., Lapidow et al., 2021; Nelson and Oakes, 2021), or more broadly, the development of a “large-scale, shared infrastructure for developmental science” (Sheskin et al., 2020).

Additionally, Rhodes et al. (2020) presented a timely analysis of their ventures into online testing using an asynchronous (unmoderated) platform both prior to and during the beginning of the pandemic, including details regarding recruitment, informed consent procedures, and parental interference. Similarly, Chuey et al. (2021) provided details regarding moderated (synchronous) testing of young children within their lab, comparing online data collection to in-person testing, and Kominsky et al. (2021) report on online testing strategies that began prior to 2020. Pre-pandemic, in contrast, there were fewer papers detailing online testing methods for young children, with notable exceptions including early reports on the *LookIt* platform (e.g., Scott et al., 2017), the *Gorilla* experiment builder (Anwyl-Irvine et al., 2020), and TheChildLab.com (Sheskin and Keil, 2018), as well as related projects designed to create a video library of developmental psychology studies (Databrary and Datavyu: Gilmore et al., 2018).

The number of publications, and the speed at which they were written, provide indirect evidence for the importance the field has placed on conducting developmental science online, and in doing so, using the best possible practices. Here, we add to our growing knowledge by providing a synthesis of current online testing practices—rather than reporting on the experiences of a single laboratory—including strategies, challenges, and successes. Inspired by a previous survey study of developmental researchers regarding in-person testing of infant participants (Eason et al., 2017), we gathered information from 159 researchers worldwide who were engaged in online testing of children age 0 to 8 years or their caregivers during 2021. We focused on this age range because these children are more likely than older children to require assistance from parents during computer use and typically need engaging, attention-holding displays, yet the range also spans a variety of possible behavioral measures such as pointing, mouse clicking, and dragging (e.g., Auxier et al., 2020; Holz and Meurers, 2021) as well as eye gaze and looking duration. We thus expected variation in responses related to our questions, which focused on (1) the motivation to conduct studies online, (2) whether the respondents research questions were modified significantly for online studies, (3) the preparation and running of studies (including challenges and successes), and (4) the recruitment and diversity of child samples (again, including challenges and successes).

The survey approach allowed us to create a general picture of these current methods in our field, highlight aspects of those methods that have the potential to impact interpretations of findings, and develop considerations for future studies. The lessons learned during the push for online research during the pandemic will have continued relevance even as restrictions on in-person research have eased: to foreshadow a finding of the present study, many researchers anticipated continuing to conduct online studies in addition to in-person studies.

## Methods

### Participants

Developmental psychology researchers studying infants and young children were invited to participate in the anonymous survey through developmental psychology email listservs (i.e., Cognitive Development Society, APA Division 7 DPNet) and direct contact via a curated list of email addresses for 81 international developmental psychology researchers. The invitation specified that those completing the survey (faculty, staff, or trainees) would be required to have completed, or planned to complete, an online study with parents/caregivers or children between the ages of 0 and 8 years. The invitation included a request to share the survey link with colleagues. If a laboratory had more than one online study, we requested that the survey be completed for each study separately. To ensure that we could identify survey responses from the same laboratory, we asked laboratories to create a unique, nonidentifying alphanumeric code to be entered in each survey.

**Figure 1:**
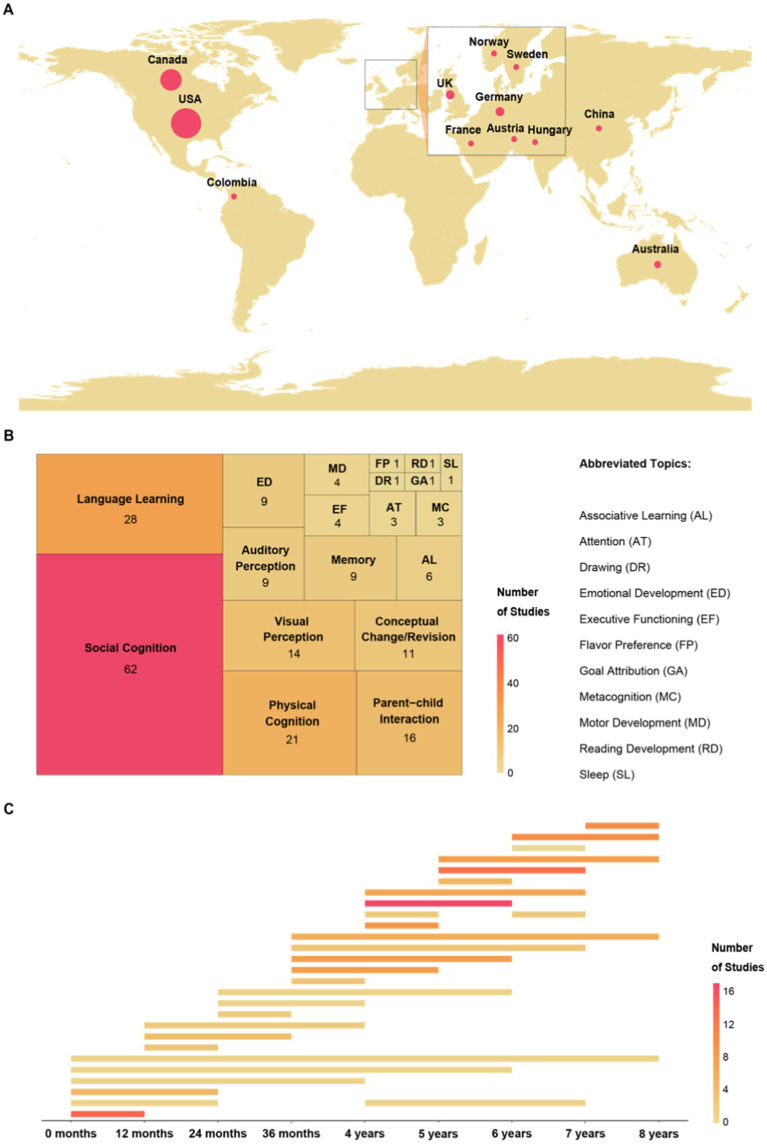
Locations of the laboratories and characteristics of the sample of online studies. **(A)** Locations of the survey respondents’ laboratories. The size of the circles represents the relative number of participating laboratories. **(B)** Values represent the frequency of endorsement of each study topic. Respondents could choose more than one topic, and thus the total number of endorsements is greater than the number of respondents. In the survey, social cognition was defined as including theory of mind, prosocial behavior, morality, and social learning, and physical cognition included number, physical causality, and tool use. In addition to the list of topics provided in the survey, respondents could choose ‘Other’ and enter a topic. **(C)** Age ranges for children tested in the online studies are depicted in one-year increments, and darker hues depict age ranges that were represented in more studies. For example, 9 studies tested children age 4–5 years, and 17 studies tested children age 4–6 years.

The sample size was determined based on the sample obtained in a previous survey of developmental psychology researchers regarding practices for in-person testing of infants (*n* = 151, Eason et al., 2017). We obtained a final sample of 159 completed survey responses (with approximately 80 responses excluded due to incomplete responses^1^). The survey responses came from 131 distinct laboratories (i.e., 17 laboratories completed the survey for more than one study). Figure 1A depicts the locations of the laboratories represented by the survey respondents. One hundred and six of the 131 laboratories were associated with universities or colleges that emphasize research and teaching at the graduate and undergraduate level (80.9%), 13 emphasized teaching at the undergraduate level with some research (9.9%), and the remaining 12 laboratories were located in private/national research institutions or medical schools (9.2%). For the 159 surveys, respondents consisted of 55 graduate students (34.6%), 26 assistant professors (16.4%), 25 postdoctoral fellows (15.7%), 20 professors (12.6%), 13 associate professors (8.2%), 9 paid laboratory staff (5.7%), 4 undergraduate students (2.5%), 2 research assistants (1.3%), and 5 (0.03%) respondents who identified as a combination of the above.

### Survey design and items

The anonymous survey was drafted, revised, and then cleared by the Queen’s University General Research Ethics Board between June 2021 and August 2021. The full survey had 65 questions, including follow-up questions for some responses, and took approximately 20 min to complete. Data were collected using the online survey platform Qualtrics from August 2021 to September 2021. The current study reports findings from the survey questions related to the focal areas noted in Introduction: (1) the motivation to conduct studies online, (2) whether the respondents’ research questions were modified significantly for online studies, (3) the preparation and running of studies, and (4) the recruitment and diversity of child samples. Data from 7 of the 65 questions are not included here (four pertained to study specifics such as start/end dates and session lengths, and three were follow-up questions pertaining to post-testing data coding) as these were not relevant for the many respondents (61%) who were reporting on studies that were still actively collecting data. The full survey and dataset are available at https://osf.io/2ntq8/.

After reading the consent form and letter of information describing the Qualtrics survey, respondents were asked to create and enter a laboratory code if their laboratory was completing more than one survey. Respondents then answered demographic questions about their laboratory’s location, their institution, and their role in the laboratory. In addition, respondents answered general questions about the nature of their online study, data collection, their motivations for conducting a study online, and whether their study included a questionnaire for parents/caregivers. This section included 10 questions in total.

Following this section, respondents were directed to two separate versions of the survey based on whether their online study involved questionnaires for parents/caregivers. If respondents’ online studies consisted entirely of a questionnaire for parents/caregivers, they were directed to a shortened version of the survey. This version consisted of 8 questions related to respondents’ general study details (i.e., study type, recruitment, participant demographics, planning), and whether respondents had concerns related to the study. If respondents’ online studies included online test sessions with child participants, they were directed to a longer version of the survey. In total, the longer section included 48 questions, some with follow-up questions depending on the response.

Many of the multiple-choice questions included an “other, please specify” option with space to describe an alternate response. Further, to account for studies that were currently running in the early stages, many questions included an “it is too soon to tell” option. At the end of the survey, respondents were asked “What else should we have asked you?” in order allow for additional information about their experiences with online testing. At completion of the survey, all respondents could opt to enter their email in a separate survey to be entered in a draw to win one of 3 $100 gift cards.

### Characteristics of the sample of online studies

The survey was designed to include online studies that consisted solely of a questionnaire for parents/caregivers (e.g., no child participants) as well as studies with child participants. But, only 11 (6.9%) studies fell into the former category, and thus only the studies with child participants will be described in this section (*n* = 148, 93.1%). Survey respondents were provided with a list of research topics for their study as well as the opportunity to write-in a topic if theirs was not reflected on the list. Respondents had the ability to choose multiple topics. The most common study topics among respondents were social cognition (including theory of mind, prosocial behavior, morality, and social learning; *n* = 62, 30.4%), language learning (*n* = 28, 13.7%), and physical cognition (including number, physical causality, and tool use; *n* = 21, 10.3%), though broad range of topics were reported. Figure 1B provides frequency values for all study topics.

In the survey, respondents selected all age ranges relevant to their study, in increments of 1 year. All ages between 0 and 8 years were represented in our sample, though more studies tested children over the age of 4 years than below (Figure 1C). Most studies tested children across multiple years, with the exception of studies testing infants between 0 and 12 months. Studies specifically testing children between 4 and 6 years were the most frequent in the sample. The survey did not ask researchers whether focal age ranges were changed to adapt to online testing.

## Results

### Motivation for, and previous experience with, online testing

At the time of the survey, the majority of respondents reported that the motivation to conduct an online study stemmed from the fact that in-laboratory testing was not possible due to the COVID-19 pandemic (*n =* 140, 88.1%). Some researchers also expressed having considered online testing prior to the pandemic, but that the pandemic pushed them to start online testing earlier than expected (*n =* 12, 7.5%). Relatively few researchers reported that their online study was planned prior to the onset of the pandemic (*n =* 7, 4.4%).

For the 11 (6.9%) of online studies in our sample that consisted solely of a questionnaire for parents/caregivers (e.g., no child participants), all but one respondent noted that their online study was motivated by the inability to conduct in-laboratory testing. Most respondents (*n* = 7) reported that this was either their first study consisting solely of a questionnaire for parents/caregivers or that it was not common for them to conduct such studies, and all of these respondents reported that a lack of familiarity with testing children in the online context was a factor relevant to planning their questionnaire study.

Most respondents who included a test session with children in their study indicated that their project was the first study they have conducted with children online (*n =* 108/148, 73.0%), and others reported that it was uncommon for them to conduct online research with children, but that they have done it before (*n =* 34/148, 23.0%). Only six respondents (4.1%) reported they conduct online studies with children regularly. Of all the respondents (*n* = 159), 125 (78.6%) reported that their research projects were intended for training purposes/degree completion.

### Modifying research questions for online testing

As a result of transitioning from in-laboratory research designs, we wanted to better understand whether researchers had to make modifications to their research question. Specifically, participants were asked whether they had to “modify a previously-planned research question/goal to suit online delivery.” Over half of respondents reported making modifications, with 20.8% (*n =* 33) making substantial modifications and 35.8% (*n =* 57) making minor modifications. The remaining respondents (*n =* 69/159, 43.4%) reported not modifying their research question.

We found that the majority of respondents did not aim to replicate previous in-laboratory projects (*n =* 104, 65.4%), though some reported that a small portion of their study was a replication attempt (*n =* 47, 29.6%). Relatively few indicated that they did aim to replicate prior in-laboratory studies (*n =* 8, 5%).

### Preparing and running online studies

#### How long did it take researchers to prepare an online study for children?

Respondents who reported testing children in their online study (rather than conducting a study with a parental questionnaire) were asked about the time it took to prepare their study. Of the 148 respondents in this category, 144 answered the question. Almost half (*n =* 68, 47.2%) reported that online study preparation took longer than typical in-laboratory studies, while the remaining 50.7% (*n* = 73) reported preparation length to be comparable to in-laboratory testing, and 2.1% (*n =* 3) reported that in-laboratory test preparation typically takes longer. For those respondents who indicated that preparation time was longer for their online study, a follow-up question asked what contributed to the preparation length, and respondents could provide more than one answer. Common reasons among these 68 respondents included creating digital stimuli (*n =* 45), pilot testing (*n =* 37), learning to use a new online platform (e.g., *Zoom* or *LookIt*; *n =* 34), learning to program/code for a new platform (*n =* 31), and choosing an online testing platform (*n =* 18).

In more specific questions about the study preparation time, posed to the full group of researchers who tested children online, we asked how long it took to choose an online study platform. Of the 147 respondents who answered, 50.3% (*n =* 74) indicated it took them less than a week to choose their platform. For the half of participants who took longer than 1 week to choose a study platform, 33.3% (*n* = 49) took less than a month, 9.5% (*n* = 14) less than 3 months, and 6.8% (*n* = 10) more than 3 months. We also asked how long it took to create the online study interface (including any stimulus creation, programming, and pilot testing, but excluding literature review, hypothesis development, and preregistration). Of the 147 respondents, 34.7% (*n* = 51) took less than a month, 33.3% (*n* = 49) took less than 3 months, 25.9 (*n* = 38) took more than 3 months, and 6.1% (*n* = 9) took less than a week. In sum, then, the time to choose and develop the online system for testing ranged from less than a month to 3 months.

#### What were commonly used testing platforms and stimulus presentation software for testing children online?

Of the respondents who were testing children online, 66.9% (*n =* 99/148) reported conducting their study using a synchronous platform in which the experimenter was present, and 24.3% (*n =* 36) reported conducting their study using an asynchronous platform. A small number of respondents reported including both synchronous and asynchronous components in their online study (*n* = 13, 8.8%).^2^ In the current sample, studies with asynchronous sessions tested proportionally more infants age 0 to 2 years (*n* = 17/49, 34.7%) than fully synchronous studies (*n* = 18/99, 18.2%), though all age ranges were represented in both types of studies.

Most of the researchers were new to conducting research with children through online platforms, and many reported that they conducted their research with platforms they were already familiar with (*n* = 99, 66.9%). One-third of respondents (*n* = 49, 33.1%) used platforms they had not used before. Eighteen (12.2%) respondents tried more than one platform before deciding on their final system. Some of these reasons for switching platforms included parental familiarity with the final platform, institutional research ethics considerations, and difficulties with a prior platform’s customizability.

**Figure 2:**
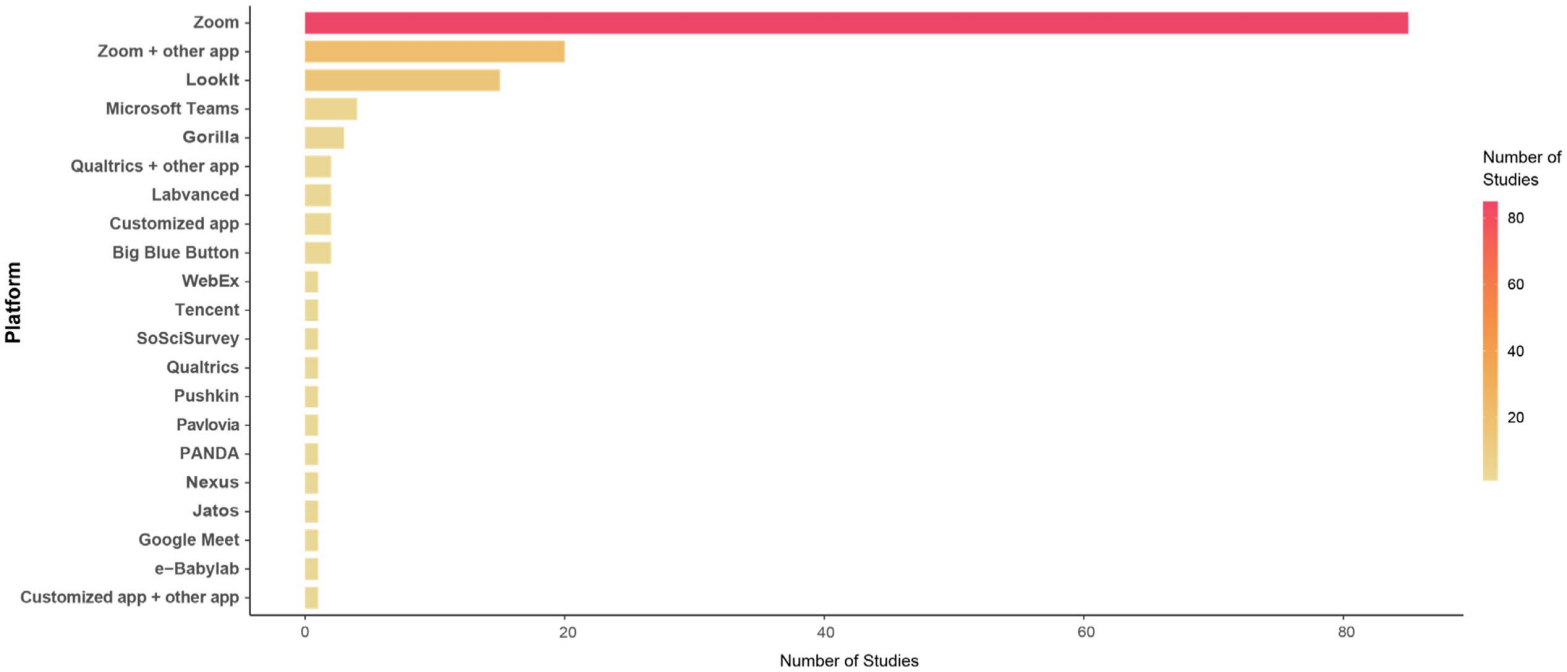
Online platforms used by studies in the sample.

Ultimately, the respondents reported used a variety of online testing platforms for their projects. The most common interfaces used for projects were *Zoom* (*n* = 105, 70.9%) and *Lookit* (*n* = 17, 11.5%), alone or in combination with other platforms. We found that *Zoom* was the most commonly used platform for synchronous studies (*n* = 90/99, 90.9%) and for studies using both synchronous and asynchronous sessions (*n* = 13, 92.3%), though less so for asynchronous studies (*n* = 3/36, 8.3%). *Lookit* was the most commonly used platform for asynchronous studies (*n* = 16, 44.4%). Figure 2 depicts all platforms used by studies in our sample, both synchronous and asynchronous.

The respondents were relatively split in opinion regarding whether they agreed with the statement, “We experienced many difficulties and challenges with this online study platform.” 40.5% of respondents (*n* = 60/148) strongly or somewhat agreed that there were many difficulties and challenges, and 52.0% strongly or somewhat disagreed. Yet, 77.0% (*n* = 114/148) of respondents strongly or somewhat agreed that they experienced benefits with their online platform, and 93.2% (*n* = 137/147) of respondents reported they would continue using their online platform in future studies.

We also asked participants about the software they used to present stimuli to participants. For example, though the testing session might be run using *Zoom* as a platform, stimuli might be displayed using presentation software, experiment-builder software, etc. For synchronous studies, the most common method was showing children a PowerPoint slide deck (*n* = 56/99, 56.6%). In asynchronous studies, the most common method was using programmed testing software to display stimuli (e.g., *Gorilla*, *PsychoPy*, *MatLab*, *jsPsych lab.js*: *n* = 20/36, 55.5%). For those using both synchronous and asynchronous elements, the most common method to present stimuli was to use a *PowerPoint* slide deck (*n* = 4/13, 30.8%) and/or programmed testing software (*n* = 4, 30.8%).

#### What behavioral measures were coded in online studies with children?

Considering only respondents who tested infants between 0 and 24 months, the most common behavioral measure was looking duration, alone or in combination with eye-gaze direction (*n* = 17/21, 80.1%). For synchronous studies with children between 2 and 8 years, the most commonly reported behavioral measure was children’s verbal responses, in combination with other measures or alone (*n* = 64/82 responses provided, 78.0%). For asynchronous studies with this age group, common measures included responses via the computer such as use of a mouse, keyboard, or touchscreen (*n* = 14/21 responses provided, 66.7%). In contrast, these computer-mediated responses were used less commonly in synchronous studies (20/82 responses provided, 24.4%).

#### Did caregivers need to provide technical support to child participants in online studies?

For synchronous and asynchronous studies, as well as studies that used both types of interaction, we considered the role of caregivers in providing technical support when computer-mediated responses were required from children age 2 years and above (*n =* 38). Researchers were asked whether all, most, many, some, or none of the caregivers had to help the child use the computer or tablet. When computer-mediated responses were required, 55.3% (*n* = 21/38) respondents reported that all, most, or many caregivers had to help the child. In contrast, for studies in which computer-mediated responses were not required, 31.1% (*n =* 23/74) of respondents reported this level of parental support.

Sixty-six respondents provided information regarding how they ensured that children could use their computer or tablet. Common strategies included practice activities, practice activities that particularly targeted use of the mouse or trackpad, and live or pre-recorded demonstrations.

#### What were common causes of data loss in online studies with children?

Survey respondents had the opportunity to respond to a series of questions regarding the amount and type of data loss experienced in online studies with children. When asked to compare data loss between online and in-laboratory studies, the majority indicated that either a similar amount of data loss occurred (*n* = 81/133 responses, 60.9%) or that more data loss occurred in their online study (*n* = 40/133 responses, 30.1%). Twelve respondents reported less data loss occurred in their online studies (9%). In Table 1, we detail specific instances of data loss, focusing on the responses to questions related to child fussiness, environmental distractions, parental interference, and internet connectivity.

**Table 1:** Sources of data loss.

*Were data lost due to…*	Fussiness		Environmental distractions (household)		Environmental distractions (researcher)		Parental interference		Internet connectivity	
	*n*	%	*n*	%	*n*	%	*n*	%	*n*	%
Yes, more than 20% of data	2	1.4	1	0.7	0	0	2	1.4	0	0
Yes, less than 20% of data	92	62.6	75	51.4	16	11	71	49	73	49.7
No	30	20.4	36	24.7	96	66.2	51	35.2	51	34.7
It is too soon to tell	23	15.6	34	23.3	33	22.8	21	14.5	23	15.6
*Total respondents*	147		146		145		145		147	

Of the respondents who coded behavior from video recordings after their test sessions (*n =* 93), 52 respondents did not experience challenges related to the video recording (55.9%), though 40 respondents did report challenges (43.0%). Common challenges that were reported were that participant data was lost due to choppy and/or low resolution of the videos, poor synchronization of audio and video, organizing and storing videos, and downloading video files.

### Recruitment and diversity

#### How were families recruited for online studies with children?

**Figure 3:**
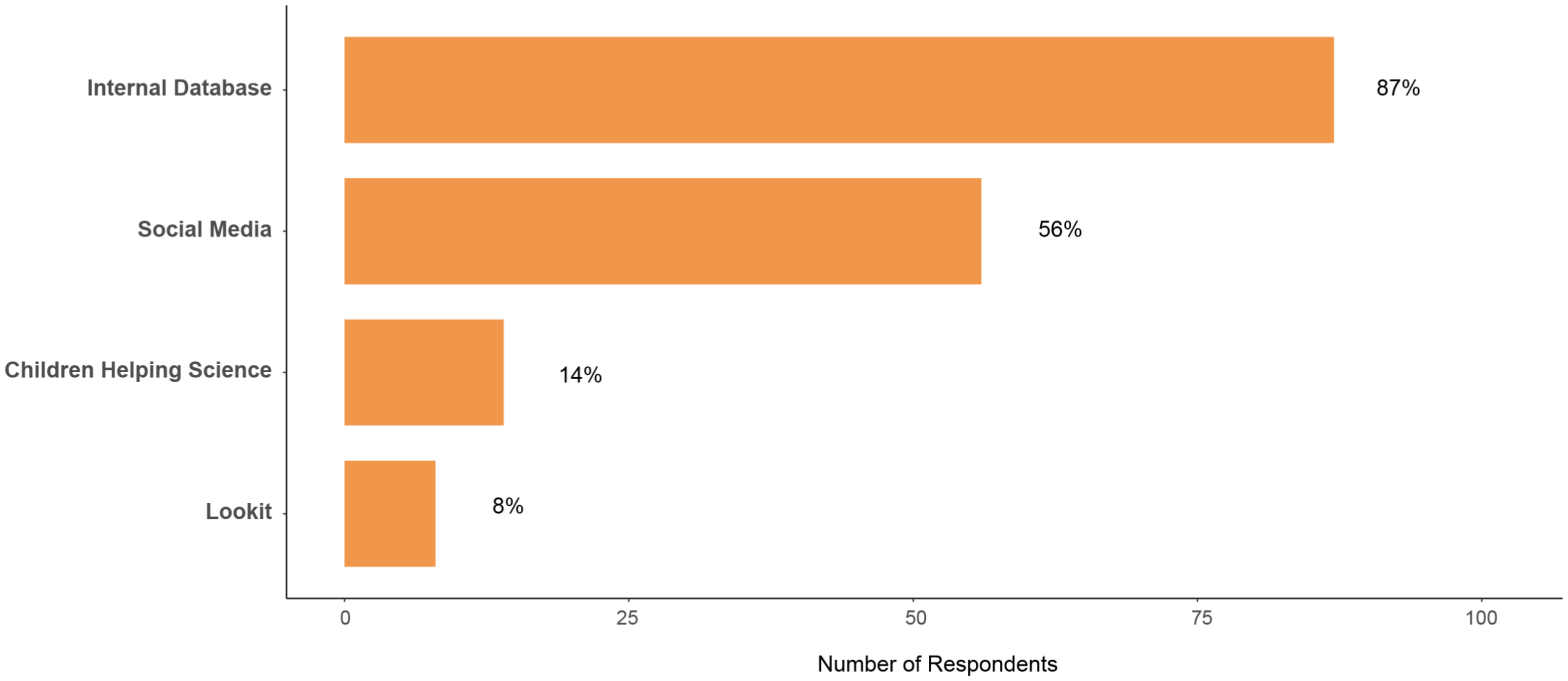
Participant recruitment methods. One hundred and forty eight respondents answered this question, and they could select more than one option. Data in this figure represent the most commonly endorsed recruitment sources.

Respondents indicated a variety of recruitment methods. When specifically asked where *most* participants were recruited from, 148 researchers provided an answer, and were allowed to select more than one option. More than half of the respondents indicated that most families were recruited from an already existing database (*n* = 87, 58.8%). Other common responses included social media (*n* = 56, 37.8%), Children Helping Science (*n* = 14, 9.5%), and Lookit (*n* = 8, 5.4%; Figure 3).

Two-thirds of respondents (*n* = 98, 66.7%) noted their recruitment methods were different than typical in-laboratory methods, while 33.3% (*n* = 49) noted their recruitment methods did not differ from in laboratory methods. Of those who responded that their recruitment methods were different than typical in-laboratory testing, common reasons included increased use of social media, fewer in-person community events, and increased use of science-specific online recruitment platforms.

#### What was the rate of testing child participants in online studies?

**Figure 4:**
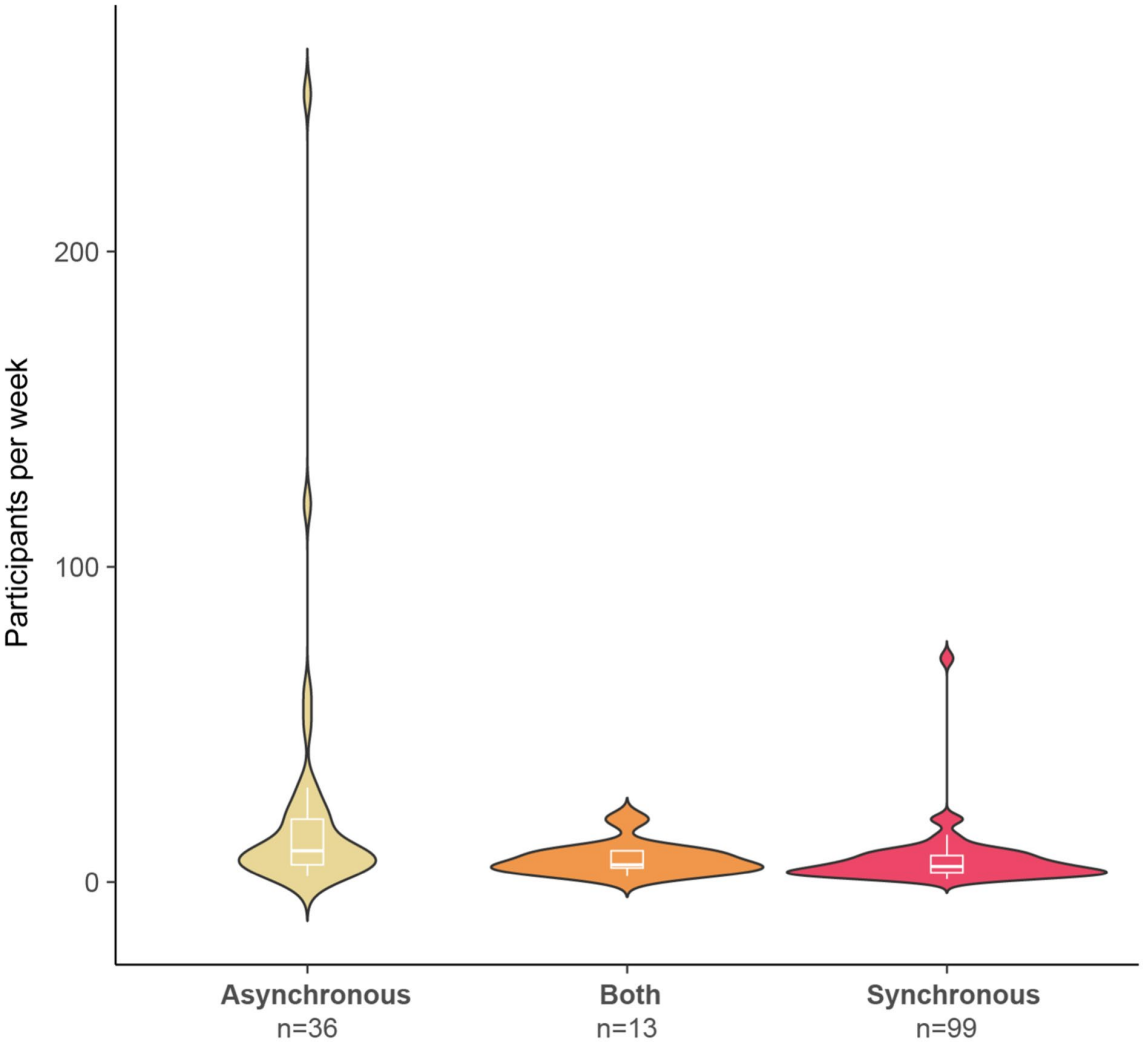
Number of children tested per week. The number of studies using each type of testing method is provided on the x-axis. The box plots within the violin plots indicate the median and interquartile range.

Researchers reported testing between 1 and 250 child participants per week online, with a mode of 10 participants per week. The highest rate of testing appeared for studies that were fully asynchronous, yet the median value of participants per week was similar across asynchronous, synchronous, and combined asynchronous/synchronous testing methods (Figure 4). For studies that were fully asynchronous or had asynchronous components, no specific age range was more likely to provide more participants per week, though studies with an above median testing rate most often had age ranges that spanned across four or more years (likely providing a larger pool to recruit from). However, this pattern was not present for studies that were fully synchronous.

Over one-half of the 148 respondents indicated that their rate of testing children online was comparable to (*n* = 32, 21.6%) or faster than (*n* = 58, 39.2%) in-laboratory studies, while 27.0% (*n* = 40) of researchers reported testing more participants per week for their in-laboratory studies. Eighteen (12.2%) respondents indicated that they could not evaluate the rate of testing at this time.

#### How diverse were child participant samples in online studies?

**Figure 5:**
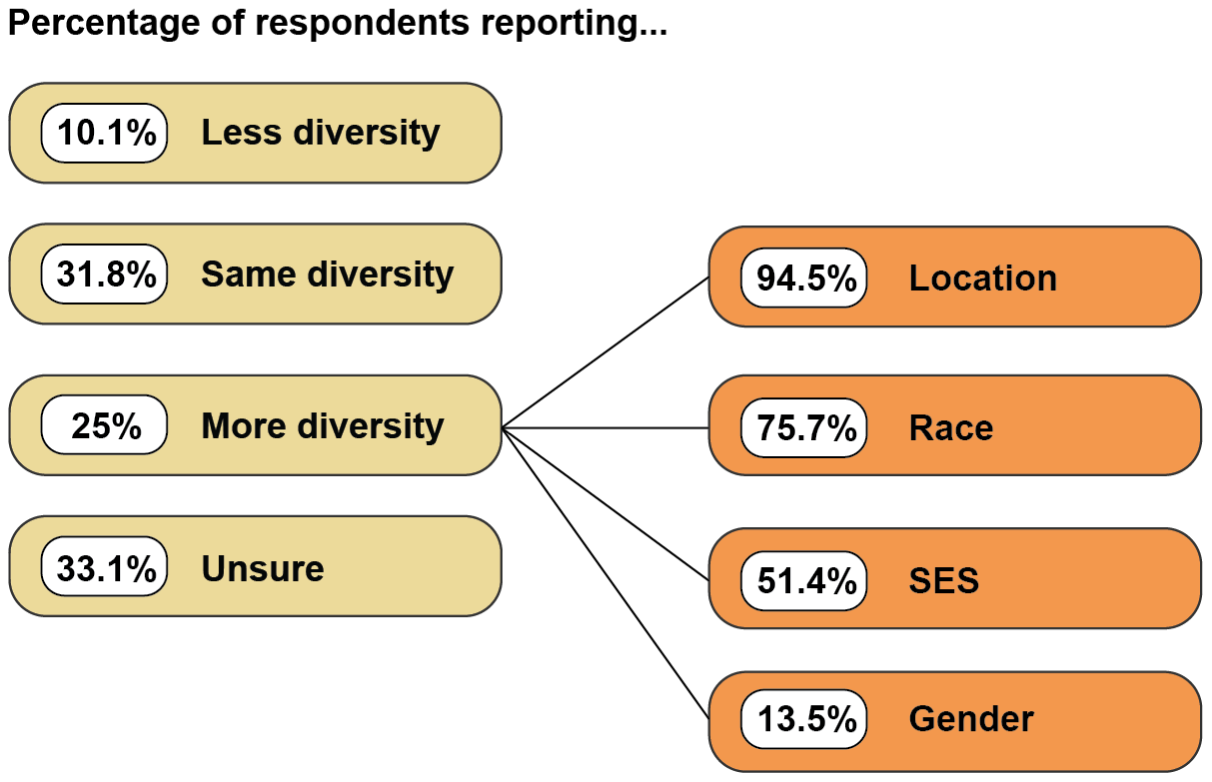
Diversity of samples compared to previous in-person studies. Participants who responded that their online sample was more diverse than samples in their typical in-person studies were asked to select all sample characteristics that were more diverse. Percentage values, thus, sum to greater than 100%.

When considering the diversity of the online study samples, 31.8% (*n* = 47) of researchers indicated their participant sample was similar in diversity to in-laboratory samples, 25.0% (*n* = 37) indicated their sample was more diverse than typical in-laboratory samples, 16.9% (*n* = 25) indicated it is too early to know, 16.2% (*n* = 24) were not sure, and 10.1% (*n* = 15) indicated the sample is less diverse than typical in-laboratory studies that test a similar age range. For the 37 respondents who noted their sample to be more diverse, a follow-up question asked them to indicate which characteristics were more diverse in the online sample, with choices including geographical location, socioeconomic status (SES), gender identification, and race. Researchers could provide more than one response. The most-commonly chosen characteristics were geographical location (*n* = 35, 94.5%) and race (*n* = 28, 75.7%; Figure 5).

### General impressions

At the end of the survey, researchers were given the opportunity to provide a response to the prompt, “What else should we have asked you? Please let us know more about your experiences with online testing of infants and young children.” All responses are included in the dataset available online, but here, we highlight comments that reflect both positive and negative evaluations of the online testing experience that were not captured in the survey questions reported above. Some respondents noted that children seemed to be more comfortable in the home environment, and that parents reported that they preferred online test sessions as they better fit into their schedules. A study testing small groups of children noted that scheduling multiple families to take part on the same day was made easier by testing online. Some researchers wondered whether the quality of data collected online would be similar to in-person testing, and others noted shorter attention spans of their child participants while online. Respondents also noted concerns related to the variability of child participant’s screen size (and distractions on the screen), and relatedly, how home screen sizes would vary from large, in-laboratory displays.

**Figure 6:**
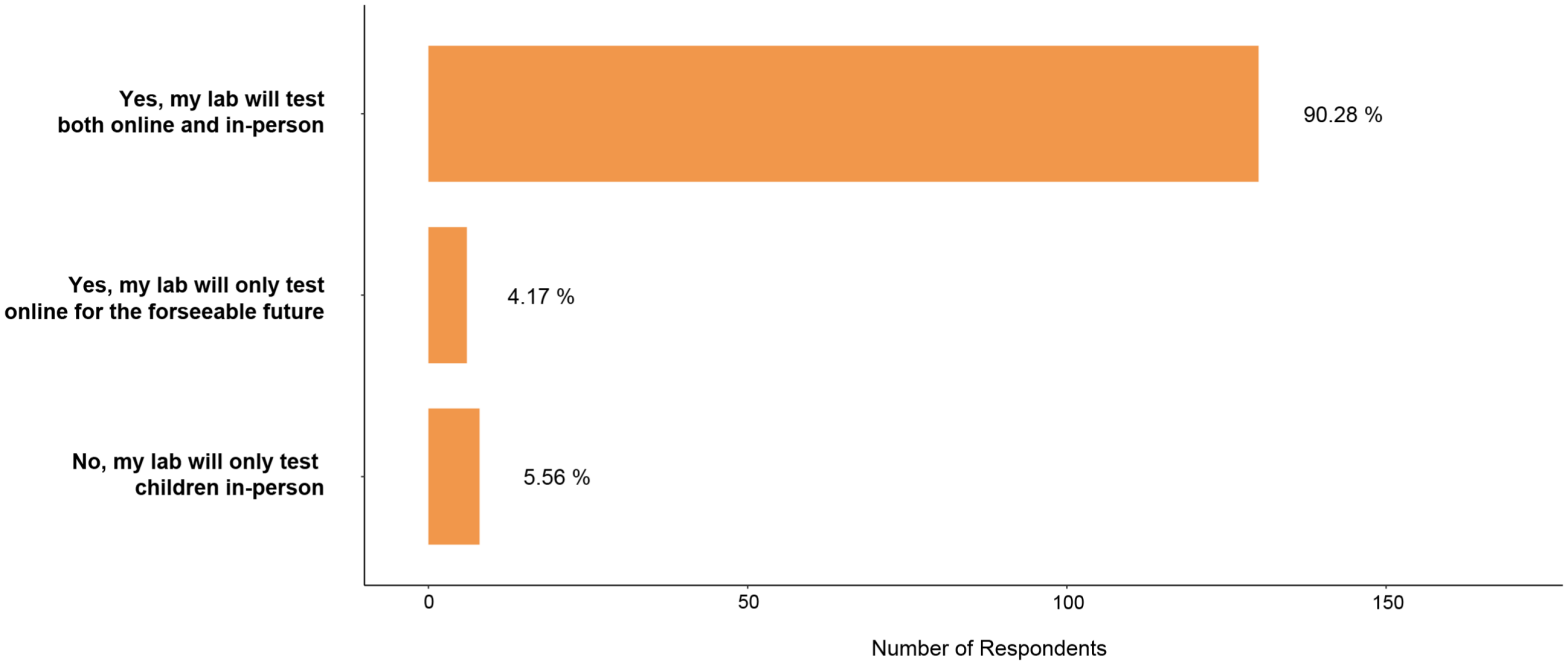
Will you continue to test children in online studies when in-person testing is possible?. One hundred and forty four survey respondents answered this question.

Yet, most respondents reported they would continue running online studies with children even when in-person research is possible (*n* = 130/144, 90.3%), with some respondents reporting they will only test in-person when in-laboratory research is possible (*n* = 8, 5.6%), and some reporting they will only test online for the foreseeable future (*n* = 6, 4.2%; Figure 6).

## Discussion

This survey study represents a snapshot of the experiences of developmental psychology researchers who conducted online studies during the COVID-19 pandemic. In most cases, this was the first time that respondents had attempted research with children and families in this manner. Additionally, as 79% of the online studies were related to training (e.g., theses), researchers presumably faced a tight timeline for degree completion, etc. Thus, we acknowledge the uniqueness of the time period in which this survey was conducted. We discuss below three notable themes that emerged from this snapshot that will be valuable to consider when planning future studies as we broaden the methodological landscape of our field to include virtual interaction.

First, though, we note some limitations of the sample to ensure that the themes that we discuss are read in context. Though the sample size was large and international, the majority of respondents were located in North America; there were only two respondents from the Global South. It is possible that the participant sample to some extent reflected the avenues we used for recruitment, which may not have reached the broader international community as intended, as well as the fact that the survey was only available in English. We also note that the survey invitation specified that respondents should be actively conducting an online study or have recently completed one; it is possible that geopolitical (e.g., war) and political (e.g., science funding) considerations resulted in the difficulty or inability in some regions to conduct research. For example, the 2021 UNESCO Science Report notes that in Brazil, “outlays by the federal agencies funding research having declined since 2015, sometimes to a remarkable extent” (Chaimovich and Pedrosa, 2021, p. 251).

Further, some of the studies that respondents reported on were ongoing, and thus, answers might have been more nuanced or opinions changed after data collection was competed. The sample may also have been limited by the availability of researchers to fill out the survey given pandemic-related time constraints (e.g., children doing remote schooling at home, preparation of online courses). Lastly, though there was a range of research topics represented in the studies described by respondents, the majority of topics fell into the broad category of cognitive development, rather than those more traditionally categorized as social–emotional development, which in turn may have limited the range of experimental approaches. Yet, the topics reported likely also reflect the age range (0 to 8 years) focused on in the survey and the current research trends in developmental psychology.

### Theme 1: technology

Challenges related to technology were consistent concerns throughout the survey. Almost half of respondents noted that it took longer to prepare for their online study than for their typical in-person studies, and reasons included having to choose an appropriate platform and, in some cases, learning specific code for the platform. We suspect that for some, this initial time investment in learning will result in faster preparation time for future studies. However, some concerns indicated that the technology itself was not as well suited for the job of online testing as the users would have hoped. A third of respondents reported losing more data in their online study than in the lab. Common reasons for data loss included internet connectivity (half of respondents), and over 40% of respondents who relied on video recordings experienced challenges.

Thus, there is a need for advancements in technology that can support online research methods for developmental psychology. We see shorter-term solutions for the challenges noted above and the need for longer-term goals. In the short term, as a field, we can continue to share knowledge. For example, Kominsky et al. (2021) offer suggestions for software that can limit the bandwidth demands when screen sharing with confederates is required and for methods that ensure smoother presentation of stimuli via software downloaded by a caregiver prior to the test session. Our own lab found success with the use of gaming software (i.e., *Construct 3*). We also encourage the continuation of workshops on online testing and the associated coding skills, perhaps modeled after preconference sessions on the use of *R* (e.g., 2019 meeting of the Cognitive Development Society), as well as continued attempts at shared infrastructure (e.g., Scott and Schulz, 2017; Sheskin et al., 2020; Lo et al., 2021).

As a longer-term goal, collaborations between researchers and the technology industry would result in the creation of platforms that do not require excessive time or technical skills to use and that specifically meet our research needs (Kominsky, 2022). These collaborations could open career paths in industry for developmental psychology graduates interested in child-computer interaction. A notable, additional outcome of shared knowledge, infrastructure, and improved functionality is the development of commonly accepted standards for online testing with children. As discussed in Zaadnoordijk et al. (2021), these standards would, in turn, be valuable to institutional research ethics committees, which often must resort to individual interpretations in the face of vague or changing regulations (e.g., regarding cloud storage of data). Though broader regulations are typically defined at the governmental level, scientific societies have a voice in this process (e.g., Litton, 2017).

### Theme 2: behavioral measures

The survey respondents reported a range of behavioral measures for children between the age of 2 and 8 years, including verbal responses, hand movements such as clapping or pointing, and computer-mediated responses such as mouse, keyboard, or touchscreen use (including using the “stamping” feature in *Zoom*). We see three points to highlight in relation to response modality. The first is related to the fact that in-person studies over the last three decades have provided good evidence that the type of response we measure may impact interpretations of task performance. Children may achieve “correct” performance when eye gaze is measured in pointing, marker placement, or verbal responses (e.g., Clements and Perner, 1994; Ruffman et al., 2001; Heine et al., 2010; Lee and Kuhlmeier, 2013), and measures of pointing actions may reveal social cognitive or spatial cognitive abilities that verbal responses do not (e.g., Crawford et al., 2000; Liszkowski et al., 2007). When faced with findings like these, researchers often consider whether cognitive mechanisms that allow for competence under one response modality may be fundamentally distinct from those that support performance under another (e.g., Karmiloff-Smith, 1992). In relation to online testing, we might consider whether, for example, indicating a choice by navigating a mouse and clicking on a red circle recruits the same processes as saying “red” or pointing to the circle. Because online testing platforms offer a range of behavioral measures to index a child’s response, we suggest (1) that detail is given in methods section regarding the measures, and (2) that care should be taken in cross-study interpretations if response modalities differ.

Second, the survey responses indicated that for synchronous studies with children between 2 and 8 years, the most reported behavioral measure was children’s verbal responses, yet for asynchronous studies with this age group, common measures included responses via the computer such as use of a mouse, keyboard, or touchscreen. Thus, when comparing results from synchronous and asynchronous studies that used different behavioral measures, researchers should consider not only the impact of the presence/absence of experimenter interaction and monitoring, but also the potential impact of the response modality.

Lastly, though computer-mediated responses offer advantages in relation to accuracy and automaticity of coding, mouse use, in particular, is challenging for younger children (e.g., Hourcade et al., 2004; Markopoulos et al., 2021). The difficulty or ease with which computer accessories are used could potentially affect the speed of responses or the willingness to continue making responses, regardless of the child’s underlying ability in relation to the research topic. Because accessory use improves with age, data that may appear to suggest a specific cognitive developmental trajectory may instead reflect, in part, a developmental trajectory for mouse expertise. Additionally, difficulty with computer-mediated responses might introduce increased need for parent involvement; in the present survey, caregiver involvement was more prevalent in studies that required computer-mediated responses than in studies that used other behavioral measures. In future online studies, it may be beneficial to consider the impact of caregiver involvement on dependent measures, particularly when computer-mediated responses are required (see Nelson et al., 2021, for an example).

### Theme 3: diversity considerations

Because online studies do not require travel to the laboratory (or other in-person venues, like museums), certain barriers to participation are likely reduced in comparison with in-person studies, such as geographic location or scheduling constraints. Online participant samples could, thus, be more diverse across many characteristics that covary with these barriers (e.g., Scott and Schulz, 2017; Zaadnoordijk et al., 2021). There was, however, little evidence of increased diversity of samples in the current survey responses, consistent with Rhodes et al. (2020). Reasons that sample diversity may not have increased for the studies reported in the survey include the fact that for most respondents, this was a first or early attempt at an online study, and the work was being conducted under unprecedented worldwide conditions. Indeed, over half of the respondents reported that most participants were recruited from their existing participant database, suggesting that new recruitment methods were not well-established.

Large scale recruitment initiatives have begun (e.g., ChildrenHelpingScience.com; KinderSchaffenWissen.de), though some barriers will likely still exist in relation to internet and web camera access. Solutions provided by Lourenco and Tasimi (2020) include providing temporary internet access to families via purchase of mobile hotspots and bringing portable computer equipment to neighborhoods via a “mobile lab” for a hybrid online/in-person approach. The authors acknowledge, though, that the financial burden on labs might make these approaches unfeasible. Additionally, we note that an additional constraint is the digital divide across countries due to a lack of infrastructure and/or internet blocking and shutdowns (UNESCO, 2022).

Other recently suggested solutions involve enhancing the relationship between a research team and their local community, which, among many possible positive outcomes, could connect researchers with underrepresented groups. Liu et al. (2021), for example, have characterized the approach that some teams have taken as the “community-engaged lab,” a collaborative network involving researchers, local businesses, children and families, museums, and community nonprofits. The formation of such a network is different than a bi-directional model wherein researchers recruit participants from the community and then disseminate findings back to the community. The network model focuses on resource and knowledge-sharing among the network members to mutual benefit. To take one example, upon hearing of the need for educational activities in the late afternoon for children at home during the first wave of COVID-19 infections, researchers in Canada at Queen’s University and the University of Toronto each offered free, weekly, virtual activity sessions over *Zoom* (e.g., “Circle Time”). The sessions were collaboratively advertised across the research groups as well as by local educators and nonprofits. The purpose of the sessions was not participant recruitment *per se*, though recruitment has occurred due to increased awareness of the research opportunities for families. Instead, the goal was to develop and maintain a mutually beneficial network. Again, though, we note that, at least for online studies, efforts such as these still do not reduce the potential barrier posed by inaccessibility to a strong internet connection.

### Conclusion

The broad goal of this survey was to create a general picture of current methods in our field, highlight aspects of those methods that have the potential to impact interpretations of findings, and develop considerations for future studies. Above, we synthesized three main themes suggested by the survey results, though we encourage others to access the data if in search of other information. Our conclusions in relation to the current period of methodological evolution in our field can be summarized as thus: (1) We should aim to create, or co-create with industry professionals, the software and systems that meet our online research needs and meet best practices for child-computer interaction. (2) We can capitalize on a rich history in developmental psychology of critically considering our behavioral measures and the conditions under which they are produced, and apply this practice to online methodologies. (3) We should continue to pursue the goal of increasing sample diversity through active outreach, international collaboration, and researcher-community networks, recognizing that the online nature of studies alone does not guarantee improved representation in our samples. Lastly, we encourage the use of future surveys of researchers to continue to reveal strengths and areas of improvement in our field.

## Data availability statement

The datasets presented in this study can be found in online repositories. The names of the repository/repositories and accession number(s) can be found at: https://osf.io/2ntq8/.

## Ethics statement

The studies involving human participants were reviewed and approved by Queen’s General Research Ethics Board. The patients/participants provided their written informed consent to participate in this study.

## Author contributions

MS, DB, SP, BH, EL, and VK designed the study and collected the data. MS, DB, SP, and VK completed the analysis and data figures and drafted and edited the manuscript. All authors contributed to the article and approved the submitted version.

## Funding

This research was supported by grants from the Natural Sciences and Engineering Research Council of Canada (NSERC) and the Social Sciences and Humanities Research Council of Canada (SSHRC) to VK. MS and DB were supported by Undergraduate Student Research Awards from NSERC.

## Conflict of interest

The authors declare that the research was conducted in the absence of any commercial or financial relationships that could be construed as a potential conflict of interest.

## Publisher’s note

All claims expressed in this article are solely those of the authors and do not necessarily represent those of their affiliated organizations, or those of the publisher, the editors and the reviewers. Any product that may be evaluated in this article, or claim that may be made by its manufacturer, is not guaranteed or endorsed by the publisher.

## Footnotes

^1^A survey was deemed to be incomplete if the survey was started but stopped prior to the final question. However, if questions were occasionally skipped at various points in the survey, yet respondents reached the final question, the survey was included in the current analyses.

^2^Of the respondents whose projects were entirely parental questionnaires, one respondent reported conducting their project synchronously (9.1%), while the majority of respondents conducted their questionnaire study asynchronously (*n* = 10, 90.9%).
